# A Cost-Effective Treatment of Spin–Orbit Couplings in the State-Averaged Driven Similarity Renormalization Group Second-Order Perturbation Theory

**DOI:** 10.3390/molecules30092082

**Published:** 2025-05-07

**Authors:** Meng Wang, Chenyang Li

**Affiliations:** Key Laboratory of Theoretical and Computational Photochemistry, Ministry of Education, College of Chemistry, Beijing Normal University, Beijing 100875, China; mwang@mail.bnu.edu.cn

**Keywords:** driven similarity renormalization group, perturbation theory, spin–orbit coupling effects, excited states

## Abstract

We present an economical approach to treat spin–orbit coupling (SOC) in the state-averaged driven similarity renormalization group second-order perturbation theory (SA-DSRG-PT2). The electron correlation is first introduced by forming the SA-DSRG-PT2 dressed spin-free Hamiltonian. This Hamiltonian is then augmented with the Breit–Pauli Hamiltonian and diagonalized using spin-pure reference states to obtain the SOC-corrected energy spectrum. The spin–orbit mean-field approximation is also assumed to reduce the cost associated with the two-electron spin–orbit integrals. The resulting method is termed BP1-SA-DSRG-PT2c, and it possesses the same computational scaling as the non-relativistic counterpart, where only the one- and two-body density cumulants are required to obtain the vertical transition energy. The accuracy of BP1-SA-DSRG-PT2c is assessed on a few atoms and small molecules, including main-group diatomic molecules, transition-metal atoms, and actinide dioxide cations. Numerical results suggest that BP1-SA-DSRG-PT2c performs comparably to other internally contracted multireference perturbation theories with SOC treated using the state interaction scheme.

## 1. Introduction

Second-order multireference perturbation theories (MRPT2s) are among the most affordable approaches to describe molecules involving strongly correlated electrons [1,2,3,4,5,6,7]. These methods start from a multi-configuration zeroth-order wave function, followed by a second-order energy correction using perturbation theory. Numerous MRPT2s have been proposed over the years, among which the complete active space perturbation theory (CASPT2) [1] and *n*-electron valence state perturbation theory (NEVPT2) [2,8] are perhaps the most commonly applied. Both CASPT2 and NEVPT2 possess a high computational scaling with respect to the number of active orbitals due to the requirement of the four-particle reduced density matrix (4-RDM) and the orthogonalization procedure of the internally contracted excited configurations. For electronic excited states, it is well documented that the state-specific CASPT2 or NEVPT2 yields artificial crossings between potential energy surfaces (PESs) of near-degenerate states [9,10]. This issue can be cured by the multi-state (MS) formulation based on the quasi-degenerate (QD) perturbation theory [9,10,11,12,13], where an effective Hamiltonian is diagonalized to allow for mixing between perturbed states. Depending on the parametrization, solving the first-order wave functions scales at least linearly with respect to the number of reference states [14].

The recently proposed state-averaged (SA) driven similarity renormalization group (DSRG) offers a state-universal approach to treat multiple near-degenerate states on an equal footing [15,16]. In the SA-DSRG formalism, the dynamical electron correlation is folded into an effective Hamiltonian via a single unitary transformation to the bare Hamiltonian. The unitary transformation depends on a time-like parameter (*s*) called the flow parameter. For a finite value of *s*, the Hamiltonian becomes band-diagonal in the Fock space, and excitations that may cause the intruder-state problem [17,18] are regularized. The ground- and excited-state energies are obtained by diagonalizing the SA-DSRG Hamiltonian in the reference space.

The SA-DSRG Hamiltonian may be approximated using perturbation theory. With a diagonal one-body, zeroth-order Hamiltonian, the resulting second-order perturbation theory (SA-DSRG-PT2) necessitates only the state-averaged 1-, 2-, and 3-RDMs. It can be shown that the computation of the 3-RDM in SA-DSRG-PT2 is unnecessary for the determination of vertical transition energies [19], making it applicable for large active spaces with more than 45 active orbitals [20]. The SA-DSRG-PT2 method provides both a continuous PES near the canonical intersection and size-intensive excitation energies [15]. Benchmarks on valence- and core-excited states [19,21,22,23] indicate that SA-DSRG-PT2 achieves an accuracy comparable to that of CASPT2 and NEVPT2 yet with reduced computational cost.

However, the application of SA-DSRG-PT2 to molecules containing heavy elements has not been extensively explored. An accurate description of these molecules requires a balanced treatment of electron correlation and relativistic effects. One of the concerning relativistic effects is spin–orbit coupling (SOC), which plays an important role in spectroscopy [24,25], magnetism [26,27], and photochemistry [28,29,30]. A rigorous treatment of the SOC effect is offered by the four-component multireference methods based on the Dirac–Coulomb or Dirac–Coulomb–Breit Hamiltonian [31,32,33,34]. Examples include the relativistic Fock-space coupled-cluster (FSCC) method [35], internally contracted multireference configuration interaction (ic-MRCI) [36], multireference Møller–Plesset perturbation theory [37], CASPT2 [38,39], NEVPT2 [36], and SA-DSRG second- and third-order perturbation theories (4c-DSRG-MRPT2/3) [40]. Nonetheless, the computational cost of these four-component methods is considerably higher than that of their non-relativistic counterparts.

More affordable methods that incorporate the SOC effect can be formulated based on a two-component relativistic Hamiltonian obtained by spin separation and the elimination of positronic degrees of freedom to the Dirac Hamiltonian [32,33,41,42,43,44]. Two-component Hamiltonians include the exact two-component (X2C) approach [45,46,47,48,49,50,51], regular approximation [52,53], the Douglas–Kroll–Hess (DKH) transformation [54,55,56,57], and the Breit–Pauli (BP) Hamiltonian [58,59]. Using a two-component Hamiltonian, a simultaneous treatment of electron correlation and the SOC effect can be achieved in a cost-efficient manner via the state-interaction spin–orbit (SISO) approach [60,61], where the Hamiltonian is diagonalized on the basis of spin-pure correlated states. Numerous methods have been developed to incorporate the SOC effect within the SISO framework, such as the density matrix renormalization group [62,63], spin–orbit multireference multistate perturbation theory [64], and QD-NEVPT2 [65,66].

In this work, we address the SOC effect in SA-DSRG-PT2 by employing a two-component Hamiltonian. The spin-free Hamiltonian (${\hat{H}}_{\mathrm{SF}}$) includes the scalar relativistic effect via the one-electron spin-free X2C (SF-X2C1e) method [46,47,48,67], while the BP Hamiltonian (${\hat{H}}_{\mathrm{SO}}$) is used for the spin-dependent contribution. The BP spin–orbit operator is commonly applied in the SISO framework [62,63,65,68]. The electron correlation effect is first addressed by transforming ${\hat{H}}_{\mathrm{SF}}$ using SA-DSRG-PT2 with RDMs obtained from the spin-pure complete active space configuration interaction (CASCI) states. Subsequently, the transformed Hamiltonian is augmented with ${\hat{H}}_{\mathrm{SO}}$ and diagonalized using the CASCI states to obtain the SOC-corrected energies. Compared to the non-relativistic SA-DSRG-PT2, additional computations for the one- and two-electron spin–orbit integrals in ${\hat{H}}_{\mathrm{SO}}$ are required. To reduce this cost, we simplify ${\hat{H}}_{\mathrm{SO}}$ to an effective one-body operator by invoking the spin–orbit mean-field (SOMF) approximation [69] with additional spin averaging [70,71]. The resulting method is termed BP1-SA-DSRG-PT2c, as it is reduced to the contracted variant of SA-DSRG-PT2 when neglecting ${\hat{H}}_{\mathrm{SO}}$, and it includes the SOC effect to the first order in perturbation theory using the BP Hamiltonian.

This article is organized as follows. In Section 2, we briefly review the standard SA-DSRG-PT2 method and formally introduce the BP1-SA-DSRG-PT2c method. The implementation of BP1-SA-DSRG-PT2c is discussed in Section 3. Then, in Section 4, we apply the BP1-SA-DSRG-PT2c scheme to compute the zero-field splittings of a few main-group elements and diatomic molecules; the excited-state energies of Cu, Ag, and Au atoms; the vertical excitation energies of [UO_2_]^+^ and [NpO_2_]^2+^; and the energy barrier for spin inversion of the single-molecule magnet (PMe_3_)_2_FeCl_3_. Finally, we summarize our findings and suggest future improvements in Section 5.

## 2. Theory

### 2.1. State-Averaged Driven Similarity Renormalization Group Second-Order Perturbation Theory

The state-averaged driven similarity renormalization group second-order perturbation theory (SA-DSRG-PT2) [15] starts by defining a vacuum under the generalized normal ordering of Mukherjee and Kutzelnigg (MK-GNO) [72]. The vacuum is an ensemble of *n* electronic states ($E\equiv \{\Psi_{0}^{\alpha },\alpha =1,2,\ldots ,n\}$) obtained from a state-averaged complete active space self-consistent field (SA-CASSCF) computation. The MK-GNO defines the operator normal ordering by requiring its ensemble average to be equal to zero. In the MK-GNO formalism, the Wick contraction generates the SA density cumulants, defined as the irreducible part of the SA-RDMs of the same order [72]. We then write the second-quantized spin-free Hamiltonian in the normal-ordered form as

(1) $${\hat{H}}_{\mathrm{SF}}={\bar{E}}_{0}+\sum_{pq}{\bar{f}}_{p}^{q}\{{\hat{a}}_{q}^{p}\}+\frac{1}{4}\sum_{pqrs}v_{pq}^{rs}\{{\hat{a}}_{rs}^{pq}\},$$

where ${\bar{E}}_{0}$ is the SA reference energy and the operator normal ordering is suggested by the curly braces. In Equation (1), we adopt the tensor notation of Kutzelnigg, where a string of spin-orbital creation and annihilation operators is given compactly as ${\hat{a}}_{rs\cdots }^{pq\cdots }=\;{\hat{a}}_{p}^{†}{\hat{a}}_{q}^{†}\cdots {\hat{a}}_{s}{\hat{a}}_{r}$. The SA Fock matrix (${\bar{f}}_{p}^{q}=h_{p}^{q}+\sum_{ij}v_{pi}^{qj}{\bar{\gamma }}_{j}^{i}$) is also introduced in Equation (1), defined using the SF-X2C1e one-electron integrals ($h_{p}^{q}$); the antisymmetrized two-electron integrals ($v_{pq}^{rs}\equiv \;\langle pq|\;|rs\rangle$); and the one-particle SA-RDM (${\bar{\gamma }}_{j}^{i}=\;\sum_{\alpha =1}^{n}\omega_{\alpha }\langle \Psi_{0}^{\alpha }\left|{\hat{a}}_{i}^{†}{\hat{a}}_{j}\right|\Psi_{0}^{\alpha }\rangle$ with $\omega_{\alpha }$ being the weight of $\Psi_{0}^{\alpha }$ in E). In this work, all states in E are assumed to be equally weighted, that is, $\omega_{\alpha }=1/n$.

In SA-DSRG [15], we perform a single unitary transformation of the Hamiltonian (${\hat{H}}_{\mathrm{SF}}$):

(2) $$\hat{H}\to \bar{H}\left(s\right)={\hat{U}}^{†}\left(s\right)\hat{H}\hat{U}\left(s\right).$$

For brevity, we ignore the subscript “SF” in Equation (2) and the following. The unitary operator $\hat{U}\left(s\right)$ depends on the so-called flow parameter s∈[0,∞) such that $\hat{U}\left(0\right)$ is an identity operator and $\bar{H}\left(0\right)=\hat{H}$. As *s* grows, the DSRG Hamiltonian $\bar{H}\left(s\right)$ becomes more and more block-diagonal. The non-diagonal elements that connect the reference state to the excited configurations gradually become zero, essentially folding in dynamical electron correlation to the diagonal part of $\bar{H}\left(s\right)$. To this end, we write $\hat{U}\left(s\right)=\exp\left[\hat{A}\left(s\right)\right]$ in terms of an anti-Hermitian operator $\hat{A}\left(s\right)=\hat{T}\left(s\right)-{\hat{T}}^{†}\left(s\right)$, where $\hat{T}\left(s\right)$ is the *s*-dependent cluster operator. The cluster amplitudes are determined by the DSRG flow equation:

(3) $${\bar{H}}_{ab\cdots }^{ij\cdots }\left(s\right)=\left[{\bar{H}}_{ab\cdots }^{ij\cdots }\left(s\right)+t_{ab\cdots }^{ij\cdots }\left(s\right)\Delta_{ab\cdots }^{ij\cdots }\right]e^{-s{\left(\Delta_{ab\cdots }^{ij\cdots }\right)}^{2}}.$$

We require the non-diagonal many-body components of $\bar{H}\left(s\right)$ [${\bar{H}}_{ab\cdots }^{ij\cdots }\left(s\right)$] to be equal to those of the source operator derived from the single-reference similarity renormalization group [73,74]. In Equation (3), $\Delta_{ab\cdots }^{ij\cdots }=\epsilon_{i}+\epsilon_{j}+\cdots -\epsilon_{a}-\epsilon_{b}$ are the Møller–Plesset energy denominators defined using semicanonical orbital energies and $t_{ab\cdots }^{ij\cdots }\left(s\right)$ are cluster amplitudes. Here, indices i,j… label the hole orbitals, while a,b… label the particle orbitals.

To derive the SA-DSRG-PT2 equations, we first partition the Hamiltonian $\hat{H}$ into a zeroth-order contribution ${\hat{H}}^{\left(0\right)}$ plus the first-order perturbation ${\hat{H}}^{\left(1\right)}=\hat{H}-{\hat{H}}^{\left(0\right)}$. The zeroth-order Hamiltonian ${\hat{H}}^{\left(0\right)}$ includes the averaged reference energy ${\bar{E}}_{0}$ and the diagonal blocks of the SA Fock operator ${\hat{F}}^{\left(0\right)}$:

(4) $$\begin{array}{cc} {\hat{H}}^{\left(0\right)} & ={\bar{E}}_{0}+{\hat{F}}^{\left(0\right)}, \end{array}$$

(5) $$\begin{array}{cc} {\hat{F}}^{\left(0\right)} & =\sum_{mn}^{C}{\bar{f}}_{m}^{n,(0)}\{{\hat{a}}_{n}^{m}\}+\sum_{uv}^{A}{\bar{f}}_{u}^{v,(0)}\{{\hat{a}}_{v}^{u}\}+\sum_{ef}^{V}{\bar{f}}_{e}^{f,(0)}\{{\hat{a}}_{f}^{e}\}. \end{array}$$

Here, the summation indices run over the core (C), active (A), and virtual (V) orbitals. In the semicanonical basis [74], the SA Fock matrices of these three blocks are diagonal such that Equation (5) can be written compactly as ${\hat{F}}^{\left(0\right)}=\sum_{p}\epsilon_{p}\{{\hat{a}}_{p}^{p}\}$ using the semicanonical orbital energies ($\epsilon_{p}={\bar{f}}_{p}^{p,(0)}$). In SA-DSRG-PT2, we approximate $\bar{H}\left(s\right)$ using perturbation theory and consider all terms up to the second order [15]:

(6) $${\bar{H}}^{\left[2\right]}\left(s\right)=\hat{H}+\left[\hat{H},{\hat{A}}^{\left(1\right)}\left(s\right)\right]+\frac{1}{2}\left[\left[{\hat{H}}^{\left(0\right)},{\hat{A}}^{\left(1\right)}\left(s\right)\right],{\hat{A}}^{\left(1\right)}\left(s\right)\right],$$

where ${\hat{A}}^{\left(1\right)}\left(s\right)$ can be determined using the first-order DSRG flow equation. It can be easily shown that the first-order cluster amplitudes are given by [15]

(7) $$\begin{array}{cc} t_{a}^{i,(1)}\left(s\right) & =\left[{\bar{f}}_{a}^{i,(1)}+\sum_{ux}^{A}\Delta_{u}^{x}{\bar{\gamma }}_{u}^{x}t_{ax}^{iu,(1)}\left(s\right)\right]\frac{1-e^{-s{\left(\Delta_{a}^{i}\right)}^{2}}}{\Delta_{a}^{i}}, \end{array}$$

(8) $$\begin{array}{cc} t_{ab}^{ij,(1)}\left(s\right) & =v_{ab}^{ij,(1)}\frac{1-e^{-s{\left(\Delta_{ab}^{ij}\right)}^{2}}}{\Delta_{ab}^{ij}}. \end{array}$$

From Equations (7) and (8), it is evident that the amplitudes are regularized in the presence of small energy denominators, thereby alleviating the associated intruder-state problem. As such, one needs to choose a reasonable *s* value so that enough dynamical correlation is included without introducing unphysical amplitudes. In practice, the value of *s* is chosen based on various benchmarks [19,22,40], especially when $\bar{H}\left(s\right)$ is approximated using the second-order perturbation theory.

To obtain the ground- and excited-state energies, we diagonalize ${\bar{H}}^{\left[2\right]}\left(s\right)$ using a set of complete active space configuration interaction (CASCI) states:

(9) $$\sum_{\beta }^{m}\langle \Psi_{0}^{\alpha }\left|{\bar{H}}^{\left[2\right]}\left(s\right)\right|\Psi_{0}^{\beta }\rangle C_{\beta }^{\alpha^{\prime }}\left(s\right)=C_{\alpha }^{\alpha^{\prime }}\left(s\right)E_{\alpha^{\prime }}\left(s\right),\;\alpha^{\prime }=1,\ldots ,m,$$

where the number of states *m* is not necessarily identical to that included in E (i.e., m≥n). When m=n, Equation (9) is known as the contracted SA-DSRG-PT2 scheme in Ref. [15], termed SA-DSRG-PT2c. For m>n, the additional states in Equation (9) provide extra degrees of freedom that relax the targeted *n* states [16]. If *m* includes all CASCI states, Equation (9) becomes equivalent to diagonalizing ${\bar{H}}^{\left[2\right]}\left(s\right)$ in the CASCI configuration space, resulting in the SA-DSRG-PT2 scheme [15]. In this work, we ignore the normal-ordered three-body contributions of ${\bar{H}}^{\left[2\right]}\left(s\right)$, which are found to yield minimal impact with respect to the single-point energies [75].

Several factors contribute to the computational cost of SA-DSRG-PT2c. First, all one- and two-electron integrals are transformed into the molecular orbital (MO) basis. This process scales as $O(N^{5})$, with *N* being the number of MOs. Second, the evaluation of ${\bar{H}}^{\left[2\right]}\left(s\right)$ necessitates the SA one-, two-, and three-body density cumulants of reference [74]. However, the three-body cumulants can be safely ignored if only the vertical transition energies are requested [19]. Assuming that these SA density cumulants are available, the cost of computing ${\bar{H}}^{\left[2\right]}\left(s\right)$ scales as $O(V^{2}C^{2}+V^{2}A^{4}+VA^{6})$, where *C*, *A*, and *V* denote the numbers of core, active, and virtual orbitals, respectively. This computational cost is generally lower than that of CASPT2 and NEVPT2, both of which involve four-particle RDMs. Lastly, building the matrix elements of Equation (9) requires the transition 1- and 2-RDMs between n(n−1) distinct pairs of reference states. For a limited number of states, the overall cost is dominated by the second step of building ${\bar{H}}^{\left[2\right]}\left(s\right)$.

### 2.2. Incorporating Spin–Orbit Coupling in SA-DSRG-PT2

To integrate SOC in SA-DSRG-PT2, we examine the following two-component relativistic Hamiltonian:

(10) $${\hat{H}}_{2c}={\hat{H}}_{\mathrm{SF}}+{\hat{H}}_{\mathrm{SO}},$$

where ${\hat{H}}_{\mathrm{SF}}$ represents the spin-free contribution that includes the scalar relativistic effect. This effect is addressed using the SF-X2C1e approach [46,47,48,67], which modifies the one-electron integrals of the non-relativistic Hamiltonian in SA-CASSCF and SA-DSRG-PT2. For the spin–orbit contribution (${\hat{H}}_{\mathrm{SO}}$) in Equation (10), we consider the BP Hamiltonian [58,59] under the spin–orbit mean-field (SOMF) approximation [69,71]:

(11) $${\hat{H}}_{\mathrm{SO}}=\sum_{\xi }\sum_{pq}F_{pq}^{\xi }{\hat{D}}_{pq}^{\xi },$$

where ξ=x,y,z indicates the component along the axis of a Cartesian coordinate system and ${\hat{D}}_{pq}^{\xi }$ represents the one-electron spin excitation operators:

(12) $$\begin{array}{c} {\hat{D}}_{pq}^{x}=\frac{1}{2}\left({\hat{a}}_{p_{↑}}^{†}{\hat{a}}_{q_{↓}}+{\hat{a}}_{p_{↓}}^{†}{\hat{a}}_{q_{↑}}\right), \end{array}$$

(13) $$\begin{array}{c} {\hat{D}}_{pq}^{y}=\frac{i}{2}\left({\hat{a}}_{p_{↓}}^{†}{\hat{a}}_{q_{↑}}-{\hat{a}}_{p_{↑}}^{†}{\hat{a}}_{q_{↓}}\right), \end{array}$$

(14) $$\begin{array}{c} {\hat{D}}_{pq}^{z}=\frac{1}{2}\left({\hat{a}}_{p_{↑}}^{†}{\hat{a}}_{q_{↑}}-{\hat{a}}_{p_{↓}}^{†}{\hat{a}}_{q_{↓}}\right). \end{array}$$

In Equation (11), the effective one-body matrix elements are given by

(15) $$F_{pq}^{\xi }=h_{pq}^{\xi }+\sum_{rs}{\bar{\Gamma }}_{rs}\left(g_{pqrs}^{\xi }-\frac{3}{2}g_{psrq}^{\xi }-\frac{3}{2}g_{rqps}^{\xi }\right),$$

where ${\bar{\Gamma }}_{rs}={\bar{\gamma }}_{s_{↑}}^{r_{↑}}+{\bar{\gamma }}_{s_{↓}}^{r_{↓}}$ is the SA-CASSCF spin-free one-particle RDM. Equation (15) is a multireference extension of the original SOMF scheme with spin averaging obtained by replacing the Hartree–Fock 1-RDM with ${\bar{\Gamma }}_{rs}$ [65,76]. The one- ($h_{pq}^{\xi }$) and two-electron ($g_{pqrs}^{\xi }$) spin–orbit integrals are defined as

(16) $$\begin{array}{cc} h_{pq}^{\xi } & =\frac{\alpha^{2}}{2}\langle \phi_{p}\left(1\right)\left|{\hat{h}}^{\xi }\left(1\right)\right|\phi_{q}\left(1\right)\rangle , \end{array}$$

(17) $$\begin{array}{cc} g_{pqrs}^{\xi } & =\frac{\alpha^{2}}{2}\langle \phi_{p}\left(1\right)\phi_{r}\left(2\right)\left|{\hat{g}}^{\xi }\left(1,2\right)\right|\phi_{q}\left(1\right)\phi_{s}\left(2\right)\rangle , \end{array}$$

with α=1/c being the fine-structure constant, along with the following operators:

(18) $$\begin{array}{cc} {\hat{h}}^{\xi }\left(i\right) & =\sum_{A}Z_{A}r_{iA}^{-3}{\left[r_{iA}\times \hat{p}\left(i\right)\right]}_{\xi }, \end{array}$$

(19) $$\begin{array}{cc} {\hat{g}}^{\xi }\left(i,j\right) & =-r_{ij}^{-3}{\left[r_{ij}\times \hat{p}\left(i\right)\right]}_{\xi }. \end{array}$$

Here, $\hat{p}\left(i\right)$ is the momentum operator of the *i*-th electron and the vector $r_{iA}$ of magnitude $r_{iA}$ is the position of electron *i* relative to nucleus *A* of charge $Z_{A}$. Vector $r_{ij}$ is defined analogously, except that it corresponds to the relative position between electrons *i* and *j*. We note that the summation indices (p,q,r,s) in Equations (11) and (15) correspond to spatial MOs, in contrast to the spin-orbital labels employed in Section 2.1.

Treating ${\hat{H}}_{\mathrm{SO}}$ as a perturbation to the spin-free Hamiltonian, we modify the SA-DSRG-PT2c effective Hamiltonian [Equation (9)] as

(20) $${\tilde{H}}_{\alpha^{\prime }\beta^{\prime }}^{\left[2\right]}\left(s\right)=\langle \Psi_{0}^{\alpha^{\prime }}\left|{\bar{H}}^{\left[2\right]}\left(s\right)+{\hat{H}}_{\mathrm{SO}}\right|\Psi_{0}^{\beta^{\prime }}\rangle ,$$

which is subsequently diagonalized to obtain the SOC-corrected energies. Note that the state labels (i.e., $\alpha^{\prime }$ and $\beta^{\prime }$) in Equation (20) indicate all spin multiplets for the *m* CASCI states considered in Equation (9). Comparing Equation (20) to Equation (9), additional computations involve building the spin–orbit integrals [Equations (16) and (17)] and the transition 1-RDMs of $\langle \Psi_{0}^{\alpha^{\prime }}\left|{\hat{D}}_{pq}^{\xi }\right|\Psi_{0}^{\beta^{\prime }}\rangle$. Nonetheless, the computational scaling remains the same. Comparing to the CASCI SISO approach [replacing ${\bar{H}}^{\left[2\right]}\left(s\right)$ with $\hat{H}$ in Equation (20)], the diagonal matrix elements and the couplings between spin-pure states are modified by the DSRG treatment of electron correlation. Because Equation (20) incorporates the SOC effect up to the first order in perturbation theory with the BP Hamiltonian, we denote this formalism as BP1-SA-DSRG-PT2c. However, for brevity, we drop the “BP1” prefix whenever possible in the following.

## 3. Implementation

The BP1-SA-DSRG-PT2c approach can be summarized as follows. We first perform an *n*-state SA-CASSCF computation to obtain the optimized MOs and the CASCI states used to construct the MK-GNO vacuum. The SA-DSRG-PT2 equations are then solved. As shown in Ref. [77], computing $\langle \Psi_{0}^{\alpha }\left|{\bar{H}}^{\left[2\right]}\left(s\right)\right|\Psi_{0}^{\beta }\rangle$ necessitates only the scalar term of ${\bar{H}}^{\left[2\right]}\left(s\right)$ and the one- and two-body components labeled completely by the active indices. These DSRG-dressed one- and two-electron terms are contracted with the transition 1- and 2-RDMs of the *m* spin-pure CASCI states, yielding the contributions to ${\tilde{H}}_{\alpha^{\prime }\beta^{\prime }}^{\left[2\right]}\left(s\right)$ [Equation (20)]. To account for the SOC effect, we compute the SOMF spin–orbit integrals [$F_{pq}^{\xi }$; Equation (15)] using the spin-traced 1-RDM (${\bar{\Gamma }}_{rs}$) of SA-CASSCF. Then, we contract these spin–orbit integrals with the transition 1-RDMs ($\langle \Psi_{0}^{\alpha^{\prime }}\left|{\hat{D}}_{pq}^{\xi }\right|\Psi_{0}^{\beta^{\prime }}\rangle$) for all spin states of the *m* CASCI states, thereby finalizing the construction of ${\tilde{H}}_{\alpha^{\prime }\beta^{\prime }}^{\left[2\right]}\left(s\right)$. Lastly, the BP1-SA-DSRG-PT2c energies are obtained by diagonalizing ${\tilde{H}}_{\alpha^{\prime }\beta^{\prime }}^{\left[2\right]}\left(s\right)$.

We implemented the above procedure in our in-house Python script. The one- and two-electron integrals of the bare Hamiltonian ($\hat{H}$) were obtained using Psi4 [78]. The open-source Forte [79] code was employed to obtain the spin-free 1-RDM of SA-CASSCF, the CASCI states used to build ${\tilde{H}}_{\alpha^{\prime }\beta^{\prime }}^{\left[2\right]}\left(s\right)$, and the SA-DSRG-PT2-transformed Hamiltonian ${\bar{H}}^{\left[2\right]}\left(s\right)$. The PySCF [80] interface of the Libcint integral library [81] was adopted to acquire the spin–orbit integrals. Notably, the two-electron contributions to $F_{pq}^{\xi }$ were computed on the fly using the JK contraction algorithm [82].

## 4. Numerical Results

### 4.1. Calibration of the Parameters of BP1-SA-DSRG-PT2c

**Figure 1:**
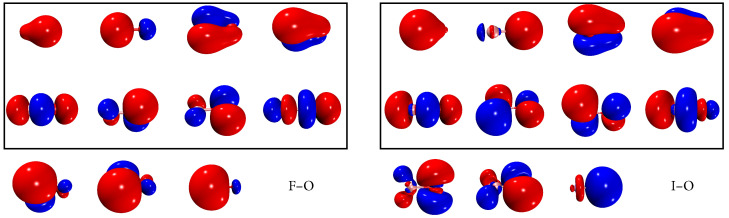
The CASSCF (13e,11o) orbitals of FO and IO, where the 8 valence orbitals are boxed.

We first assess the accuracy of BP1-SA-DSRG-PT2c on the spin–orbit zero-field splitting (ZFS) in the ^2^Π ground state of FO and IO molecules by adjusting the active space, the number of CASCI states (*m*), and the DSRG flow parameter (*s*). Two active spaces were examined. The smaller one includes all the valence orbitals of oxygen and the halogen atom, resulting in an active space of 13 electrons in 8 orbitals, denoted as (13e,8o). The larger (13e,11o) active space augments the above eight active orbitals with an additional set of π and one σ anti-bonding orbital (see Figure 1). In the SA-CASSCF computation, only the doubly degenerate ground state is taken into account, as our focus is the ZFS of the ^2^Π ground state. Unless otherwise noted, we employed the uncontracted (unc) ANO-RCC basis set [83] throughout this work. Moreover, the density-fitted two-electron integrals were assumed in this work, and the auxiliary basis set was automatically generated [84].

**Figure 2:**
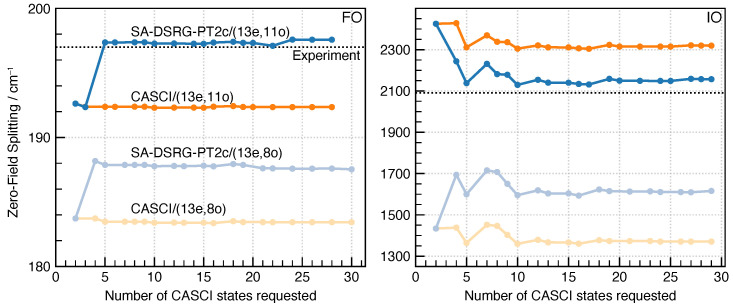
Zero-field splittings in the ground ^2^Π state of FO and IO molecules obtained using BP1-SA-DSRG-PT2c/unc-ANO-RCC with s=0.5$E_{h}^{-2}$.

Figure 2 plots the ground-state ZFSs of FO and IO molecules computed using SA-DSRG-PT2c, along with those of CASCI, where ${\bar{H}}^{\left[2\right]}\left(s\right)$ is replaced by the bare spin-free Hamiltonian ($\hat{H}$) in the SISO diagonalization [Equation (20)]. The DSRG treatment of electron correlation generally brings the ZFS result closer to the experimental value. Comparing the two active spaces, the larger active space offers a better starting point for the DSRG correction to the Hamiltonian. We also tried to perform the small active-space SA-DSRG-PT2c/(13e,8o) computations using the CASSCF (13e,11o) orbitals. The resulting ZFS values appear identical to those of SA-DSRG-PT2c/(13e,11o), indicating that the SOC matrix elements are sensitive to the quality of valence orbitals. A fast convergence of the ground-state ZFS is observed with respect to the number of CASCI states (*m*), where m∼10 (doublet states) is enough to obtain a converged result. This behavior is expected because the ground-state ZFS is mainly determined by the coupling within the ground ^2^Π multiplet, and that with other states provides a secondary correction. Both the DSRG transformation and the SOMF integrals assume the SA-CASSCF RDMs, resulting in identical matrix elements (${\tilde{H}}_{\alpha^{\prime }\beta^{\prime }}^{\left[2\right]}$) between the ground ^2^Π multiplet.

**Figure 3:**
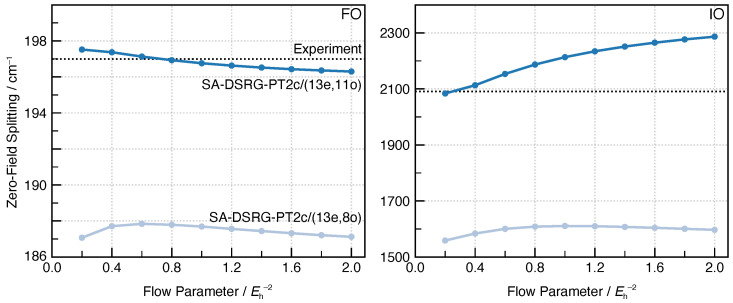
Zero-field splittings in the ground ^2^Π state of FO and IO molecules obtained using BP1-SA-DSRG-PT2c/unc-ANO-RCC with varying *s* values. The number of CASCI states is m=15 and m=16 for FO and IO, respectively.

In Figure 3, we plot the ground-state ZFS of FO and IO as a function of *s* while keeping the *m* value fixed at 15 and 16 for FO and IO, respectively. For the (13e,8o) active space, the ZFS value remains largely constant in the tested range of s∈[0.2,2.0] $E_{h}^{-2}$, differing by no more than 2 % of the ZFS value predicted at s=0.5 $E_{h}^{-2}$. For the larger (13e,11o) active space, better agreement with the experimental value is obtained using a small value of *s*, which is consistent with our previous benchmark on vertical excitation energies against theoretical best estimates [19]. As a result, we adopt s=0.5 $E_{h}^{-2}$ in the following computations. This value of *s* is more or less universal and has also been shown to yield reasonably accurate results on global potential energy surfaces [15] and vertical transition energies [19].

### 4.2. Main-Group Atoms and Diatomic Molecules

We now focus on the ZFS in the doublet ground state of a few main-group elements and diatomic molecules, where both theoretical and experimental reference data are available. These open-shell species include the group 13 and 17 atoms, the group 14 and 16 hydrides, and the halogen monoxides. We directly took the molecular geometry, the active space, and the number of CASCI states in the SISO diagonalization from Ref. [66]. Two sets of SA-CASSCF orbitals were employed. The first was obtained by averaging only the ground doublet state (i.e., n=2), while the other included all states used in SISO (i.e., n=m). The unc-ANO-RCC-VTZP basis set [83] was adopted for the group 14 hydrides to be consistent with that used in QD-NEVPT2 [66].

**Figure 4:**
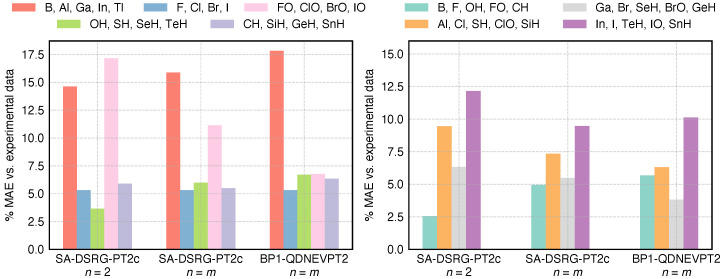
Mean absolute error (MAE, in %) for the zero-field splittings of the main-group elements and diatomic molecules relative to the experimental data, classified by according to the group (**left**) and the period (**right**) of the periodic table. See Table 1 for the ZFS value of each individual system.

The ZFS results of SA-DSRG-PT2c are reported in Table 1. The use of two different sets of SA-CASSCF orbitals in SA-DSRG-PT2c yields fairly consistent results, which differ by 3.6%, on average, from the experimental value. The most striking difference is observed for the IO molecule (278 ${\mathrm{cm}}^{-1}$, 13.3%), where the coupling between the ground ^2^Π multiplet is found to be stronger for n=m than that for n=2. Significant deviations are observed between BP1-SA-DSRG-PT2c and 4c-DSRG-MRPT2 for Ga and Cl atoms, which is likely caused by the error introduced in the BP Hamiltonian for the late period 3 elements of the periodic table [66], while the comparable result of Br is likely fortuitous. In Figure 4, we compare the BP1-SA-DSRG-PT2c results against those of BP1-QD-NEVPT2 by plotting the percentage of the mean absolute error (MAE) relative to the experimental data. When using the same set of SA-CASSCF orbitals, SA-DSRG-PT2c and QD-NEVPT2 offer similar accuracy, and the MAE of each period differs by no more than 1.5 %. We note that only 1- and 2-RDMs are required to obtain the ZFS of SA-DSRG-PT2c, making it a cheaper alternative to the QD-NEVPT2 method.

### 4.3. Transition-Metal Elements: Cu, Ag, and Au

In this section, we explore the SOC effect in the excited ^2^D term of Cu, Ag, and Au atoms using BP1-SA-DSRG-PT2c. The ^2^D term involves strongly correlated d electrons resulting from the $nd^{9}\left(n+1\right)s^{2}$ (n=3,4,5) configuration. Consistent with previous work [60,62], the active orbitals consist of the valence nd and (n+1)s orbitals and an additional set of (n+1)d orbitals, leading to the (11e,11o) active space. Only the ground ^2^S and the lowest ^2^D states were averaged in SA-CASSCF and to construct the SA-DSRG-PT2 Hamiltonian with equal weights (0.5 for ^2^S and 0.5 for ^2^D).

Table 2 reports the SA-DSRG-PT2c predictions compared against those of CASPT2 and DMRG, as well as experimental values. The *J*-averaged excitation energies of SA-DSRG-PT2c are significantly improved over those of SA-CASSCF, with the mean absolute error (relative to experiments) reduced from 0.41 to 0.05 eV. We mention that the ZFS values of SA-DSRG-PT2c are identical to those of SA-CASSCF because only the ground ^2^S and ^2^D states are considered in the SISO step. A large deviation (0.21 eV) on the ZFS of Au is observed between SA-DSRG-PT2c and CASPT2. Two aspects may contribute to this difference. The major factor is the use of state-specific CASSCF orbitals in the CASPT2 computations [60] versus the SA-CASSCF orbitals in SA-DSRG-PT2c. To this end, extending the active space in SISO improves accuracy with the state-averaged orbitals, as suggested by the DMRG data in Table 2. The other factor stems from the use of the BP Hamiltonian in SA-DSRG-PT2c, which is considered a low-*Z* approximation. Nonetheless for Au, this effect is found to be small (∼0.01 eV) [66].

### 4.4. Actinide Oxides: [UO_2_]^+^ and [NpO_2_]^2+^

Lastly, we compute the lowest ^2^Φ_u_ and ^2^Δ_u_ terms of [UO_2_]^+^ and [NpO_2_]^2+^ molecules. In these systems, the spin–orbit coupling effect appears to be vital and interleaves the electronic states as ^2^Φ_5/2u_ < ^2^Δ_3/2u_ < ^2^Φ_7/2u_ < ^2^Δ_5/2u_ [66,93,94,95,96,97]. For both molecules, we employed the (7e,10o) active space and the ANO-RCC-VTZP basis set [83]. Only the lowest ^2^Φ_u_ and ^2^Δ_u_ terms were included in SA-CASSCF and SA-DSRG-PT2, with equal weights, while 25 doublet states were considered in the SISO procedure. The geometries of [UO_2_]^+^ and [NpO_2_]^2+^ were taken from Ref. [66] with $r_{U-O}=1.802$ Å and $r_{\mathrm{Np}-O}=1.70$ Å, respectively.

The excitation energies of [UO_2_]^+^ and [NpO_2_]^2+^ are reported in Table 3 and Table 4, respectively. We compare the SA-DSRG-PT2c results to those of QD-NEVPT2, single-state and extended multi-state (XMS) CASPT2, intermediate Hamiltonian FSCC theory with singles and doubles (IHFSCCSD), and the stochastic heat-bath configuration interaction (SHCI). We take the latter two as theoretical best estimates, as they adopt a spinor reference state. Nonetheless, the virtual space was truncated in IHFSCCSD and SHCI computations. For [UO_2_]^+^ (Table 3), all methods predict a similar vertical transition energy (VTE) of ^2^Δ_3/2u_, deviating from the experimental value by no more than 0.03 eV. The ZFS values of IHFSCCSD are generally lower than those based on SISO. For example, the SA-DSRG-PT2c values of ZFS(^2^Φ_7/2u_ − ^2^Φ_5/2u_) and ZFS (^2^Δ_5/2u_ − ^2^Δ_3/2u_) are 0.14 and 0.17 eV larger than those of IHFSCCSD, respectively. As pointed out in Ref. [66], the accuracy of QD-NEVPT2 can be improved by treating both electron correlation and SOC with the DKH Hamiltonian at the second order in perturbation theory. Indeed, better agreement with IHFSCCSD is observed for DKH2-QD-NEVPT2 than BP1-QD-NEVPT2.

For [NpO_2_]^2+^ (Table 4), the SA-DSRG-PT2c values are most consistent with those of CASPT2 and differ by 0.01 eV, on average. Taking the SHCI results as the reference, the SA-DSRG-PT2c method overestimates the ZFS of ^2^Φ_u_ and ^2^Δ_u_ by 0.13 and 0.11 eV, respectively. Similar to [UO_2_]^+^, a superior accuracy is obtained by QD-NEVPT2 when SOC is treated at the second order with the DKH Hamiltonian. This observation suggests that SA-DSRG-PT2c may also benefit from a consistent second-order description of electron correlation and spin–orbit coupling.

### 4.5. Mononuclear Single-Molecule Magnet: (PMe_3_)_2_FeCl_3_

**Figure 5:**
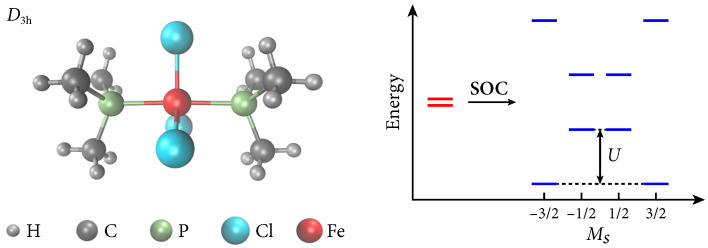
The molecular structure of (PMe_3_)_2_FeCl_3_ (**left**) and an energy-splitting sketch of the doubly degenerate quartet ground state under spin–orbit couplings (**right**).

Finally, we show that the current formalism can be applied to medium-sized molecules. In particular, we consider the energy barrier (*U*) for spin inversion of (PMe_3_)_2_FeCl_3_, which originates from the ZFS of the doubly degenerate quartet ground state [100] (see Figure 5). We took the molecular geometry from Ref. [101], which was optimized using CAM-B3LYP/def2-TZVP. Following Ref. [101], we adopted the CAS(9e,7o) active space, which includes the set of 3d orbitals of Fe and two 3p orbitals of P atoms. Four quartet states and ten sextet states were averaged in SA-CASSCF and SA-DSRG-PT2, with equal weights, and the same number of states was used in the SISO procedure. The unc-cc-pVTZ basis set [102,103] was used, leading to 966 basis functions.

The experimental effective barrier height obtained by fitting the magnetic relaxation time versus inverse temperature appears to be $U_{\mathrm{eff}}=81$ ${\mathrm{cm}}^{-1}$ [100]. An alternative value may be calculated using the experimentally fitted axial ZFS (D=−50 c$m^{-1}$ [100]) as $U=|D|(S^{2}-1/4)=100$ c$m^{-1}$ [104]. The SA-DSRG-PT2c prediction of the barrier height is U=103 c$m^{-1}$, showing an excellent agreement with the alternative experimental value. The SA-DSRG-PT2c value is also comparable to that of CASPT2 (81 c$m^{-1}$) [101]. This deviation is likely caused by the use of different spin–orbit operators, where the CASPT2 computations adopted the first-order DKH Hamiltonian.

Before we conclude, we mention the wall time and resources used in the BP1-SA-DSRG-PT2c computation on (PMe_3_)_2_FeCl_3_. From a theoretical perspective, the BP1-SA-DSRG-PT2c scheme is directly comparable to that of SOMF-QDNEVPT2 [65]. Given that the two approaches employ the same SISO procedure for SOC, the difference in computational cost results from the underlying methods, namely SA-DSRG-PT2c and QDNEVPT2. For SA-DSRG-PT2c, forming the Hamiltonian ${\bar{H}}^{\left[2\right]}\left(s\right)$ took 155 s using 50 threads of the AMD EPYC 7H12 chip, and the actual memory usage was around 4 GB. Using the same setting, the QDNEVPT2 computation took 3045 s because the first-order wave function of each state had to be solved individually. This comparison highlights the computational efficiency of SA-DSRG-PT2c.

## 5. Conclusions

In conclusion, we have presented a cost-effective approach to incorporate spin–orbit couplings in the state-averaged driven similarity renormalization group second-order perturbation theory. This approach belongs to the state-interaction spin–orbit framework, where any two-component Hamiltonian can be used. In this work, the scalar relativistic effect is addressed by the one-electron exact two-component method, and the Breit–Pauli Hamiltonian is adopted for the spin–orbit part with the mean-field approximation. The resulting BP1-SA-DSRG-PT2c method inherits the computational scaling from its non-relativistic counterpart, where the one- and two-body density cumulants of the reference wave functions are necessary to obtain vertical transition energies. As such, the BP1-SA-DSRG-PT2c approach is computationally more favorable than other internally contracted second-order multireference perturbation theories, which require the more expensive computation of higher-body reduced density matrices of the reference wave function. Numerical tests of zero-field splittings and excitation energies of atoms and small molecules show that SA-DSRG-PT2c yields an accuracy comparable to that of QD-NEVPT2 [65] and CASPT2 [60].

We note that the BP-SA-DSRG-PT2c formalism is not expected to perform well for heavy elements due to the use of a BP spin–orbit operator. Indeed, a deterioration of accuracy is clearly observed moving down the periodic table (see Table 1 and Table 2). For actinide oxides, the deviation of ZFS relative to the four-component method becomes significant (>0.1 eV). However, four-component methods remain computationally demanding, and electrons and/or spinors may be truncated to make the computation feasible (see Table 3 and Table 4). To this end, two-component methods provide a balance between accuracy and affordability. Given the increasing error in the BP Hamiltonian for elements beyond the third row of the periodic table, it is plausible to replace it with other more robust spin–orbit operators [44,66,105], including X2C and the first-order DKH Hamiltonians. For heavy elements, it may also be beneficial to treat the spin–orbit contribution directly as a first-order perturbation to build the DSRG Hamiltonian, thereby achieving a consistent treatment of electron correlation and SOC. The results of gold atoms reveal the importance of using state-specific orbitals, which remains unexplored for multi-state or SA-DSRG [75]. We believe that this work paves the way for these interesting future developments.

## Figures and Tables

**Table 1:** Spin–orbit zero-field splitting (ZFS, in ${\mathrm{cm}}^{-1}$) in the ^2^P ground term of atoms and the ^2^Π ground term of diatomic molecules.

System	SA-DSRG-PT2c ^a^		QD-NEVPT2 ^b^	4c-DSRG-MRPT2 ^c^	Experiment	
	n=2	n=m			ZFS	Ref.
B	14.7	14.9	15.0	13.99	15.3	[85]
Al	104.0	105.8	107.6	106.70	112	[85]
Ga	844.0	865.7	887.4	743.28	826	[85]
In	2459.6	2470.5	2560.8		2213	[85]
Tl	11,591.0	12,065.6	12,475.8		7793	[85]
F	401.5	401.5	401.5	384.70	404	[85]
Cl	789.7	789.7	789.7	867.69	882	[85]
Br	3574.4	3574.4	3574.4	3546.46	3685	[85]
I	8150.1	8150.1	8150.0		7603	[85]
CH	27.8	28.5	29.0		27	[86]
SiH	131.2	131.9	128.0		142	[86]
GeH	880.4	864.1	864.1		892	[86]
SnH	2433.1	2311.8	2286.3		2178	[86]
OH	139.8	149.2	152.5		139	[86]
SH	354.7	374.4	375.6		377	[86]
SeH	1742.9	1832.7	1836.7		1763	[87]
TeH	4080.9	4271.0	4293.5		3816	[88]
FO	187.8	180.0	180.0		197	[89]
ClO	270.0	280.3	299.7		322	[90]
BrO	741.4	853.2	961.9		975	[91]
IO	1593.3	1871.7	2303.8		2091	[92]

^a^ This work: first-order SOC using the BP Hamiltonian, s=0.5 $E_{h}^{-2}$. ^b^ Ref. [66]: first-order SOC using the BP Hamiltonian. ^c^ Ref. [40]: unc-cc-pVTZ basis set, s=0.24 $E_{h}^{-2}$.

**Table 2:** Excitation energies (relative to ^2^S, in eV) for the lowest ^2^D term of Cu, Ag, and Au atoms.

System	State	SA-CASSCF ^a^	SA-DSRG-PT2c ^a^	CASPT2 ^b^	DMRG ^c^	Exp. ^d^
Cu	^2^D (no SOC)	1.66	1.36			1.49
	^2^D_5/2_	1.55	1.26	1.43	1.31	1.39
	^2^D_3/2_	1.81	1.52	1.69	1.57	1.64
	ZFS	0.26	0.26	0.26	0.26	0.25
Ag	^2^D (no SOC)	4.46	3.98			3.97
	^2^D_5/2_	4.23	3.75			3.75
	^2^D_3/2_	4.80	4.33			4.30
	ZFS	0.58	0.58			0.55
Au	^2^D (no SOC)	2.30	1.74	1.58	1.62	1.74
	^2^D_5/2_	1.61	1.05	0.97	1.02	1.14
	^2^D_3/2_	3.33	2.77	2.49	2.55	2.66
	ZFS	1.72	1.72	1.51	1.53	1.52

^a^ This work: the SA-CASSCF orbitals, the unc-ANO-RCC basis set, the BP Hamiltonian for SOC, and s=0.5 $E_{h}^{-2}$. ^b^ Ref. [60]: the state-specific CASSCF orbitals, the ANO-L basis set, and the DKH Hamiltonian for SOC. ^c^ Ref. [62]: the SA-CASSCF orbitals, the ANO-RCC-VTZP basis set without g functions, the (19e,45o) active space for Cu, the (43e,57o) active space for Au, and the BP Hamiltonian for SOC. ^d^ Ref. [85].

**Table 3:** Vertical transition energies (relative to ^2^Φ_5/2u_, in eV) of [UO_2_]^+^.

Method	^2^Δ_3/2u_	^2^Φ_7/2u_	^2^Δ_5/2u_
SA-DSRG-PT2c ^a,b^	0.35	0.85	0.99
QD-NEVPT2 ^a,c^	0.36	0.80	0.98
CASPT2 ^a,d^	0.32	0.83	0.98
QD-NEVPT2 ^a,e^	0.35	0.76	0.95
IHFSCCSD ^f^	0.34	0.71	0.81
Exp. ^g^	0.33		

^a^ The (7e,10o) active space and the ANO-RCC-VTZP basis set were employed. ^b^ This work: SOC treated at the first order using the BP Hamiltonian, s=0.5$E_{\;h}^{-2}$. ^c^ Ref. [66]: SOC treated at the first order using the BP Hamiltonian. ^d^ Ref. [94]: SOC treated at the first order using the DKH Hamiltonian, $r_{U-O}=1.809$ Å. ^e^ Ref. [66]: SOC treated at the second order using the DKH Hamiltonian. ^f^ Ref. [98]: four-component computations with the Dirac–Coulomb Hamiltonian; the Faegri basis set for U and the unc-cc-pVTZ basis set for O; 24 electrons ($6p^{6}5f^{3}6d^{1}7s^{2}$ of U and $2s^{2}2p^{4}$ of O) were correlated; spinors with energies larger than 6 a.u. were excluded; $r_{U-O}=1.739$ Å. ^g^ Ref. [99].

**Table 4:** Vertical transition energies (relative to ^2^Φ_5/2u_, in eV) of [NpO_2_]^2+^.

Method	^2^Δ_3/2u_	^2^Φ_7/2u_	^2^Δ_5/2u_
SA-DSRG-PT2c ^a,b^	0.38	1.02	1.16
QD-NEVPT2 ^a,c^	0.45	1.00	1.15
XMS-CASPT2 ^a,d^	0.45	0.98	1.18
CASPT2 ^a,e^	0.38	1.00	1.15
QD-NEVPT2 ^a,f^	0.45	0.94	1.11
IHFSCCSD ^g^	0.44	0.90	1.11
SHCI ^h^	0.43	0.89	1.10

^a^ The (7e,10o) active space and the ANO-RCC-VTZP basis set were employed. ^b^ This work: SOC treated at the first order using the BP Hamiltonian, s=0.5 $E_{h}^{-2}$. ^c^ Ref. [66]: SOC treated at the first order using the BP Hamiltonian. ^d^ Ref. [96]: SOC treated at the first order using the DKH Hamiltonian. ^e^ Ref. [94]: SOC treated at the first order using the DKH Hamiltonian, $r_{\mathrm{Np}-O}=1.712$ Å. ^f^ Ref. [66]: SOC treated at the second order using the DKH Hamiltonian. ^g^ Ref. [93]: four-component computations with the Dirac–Coulomb Hamiltonian; the double-ζ basis set of Dyall for Np and the unc-cc-pVTZ basis set for O; 24 electrons were correlated; spinors with energies larger than 6 a.u. were excluded; $r_{\mathrm{Np}-O}=1.701$ Å. ^h^ Ref. [97]: spinor computations with the X2C atomic mean-field Hamiltonian; the unc-ANO-RCC basis set for Np and the unc-cc-pVTZ basis set for O; the (13e,60o) active space.

## Data Availability

Data is contained within the article.
